# Strategies applied by different arts and cultural organizations for their audience development: A comparative review

**DOI:** 10.1016/j.heliyon.2023.e15835

**Published:** 2023-04-30

**Authors:** Nada Saad Alnasser, Lim Jing Yi

**Affiliations:** Universiti Sains Malaysia, Malaysia

**Keywords:** Audience development, Strategy, Museum, Theatre, Library, Music

## Abstract

A set of mutations in social, educational, and political roles as well as in the economic contexts of the “*arts and cultural organizations*” over the past decades have highlighted their need to work on their relationship with audiences. The aim of this paper is to investigate the current debated available in the literature on the “*audience development*” in four types of “*arts and cultural organizations*”, namely, museums, theatres, libraries and music institutions, with the goal of identifying and comparing the applied strategies by these organizations for their audience development. An exploratory literature review was conducted using the databases: Google Scholar and Semantic Scholar, in addition to the websites of concerned organizations. Nine strategies of “audience development” were identified: Digital Technology, Partnerships, Physical space development, education, audience segmentation, public engagement, audience research and marketing.

## 1 Introduction

Arts and cultural organizations, such as museums, music institutions, libraries, theatres, cultural centers and cinemas, have different tasks in society, and in different eras, they deal with multiple topics such as social traditions, religion, science, politics, and history, but they can also be just for the entertainment of the audience. These organizations can participate in building audience knowledge of art and culture related to their own society and to that of the other societies and cultures in the world, thus broadening audiencehorizons. In this way, “*arts and cultural organizations*” promote the intercultural dialogue and the cultural diversity. The roles of these organizations arecontinually developing. However, in order to be remarkable in our actual changing world at different levels such as social, technological, economical and more recently regarding Covid-19 pandemic and the resulted confinement, they need to invite their audience to think about different ideas and their concern to social issues in a broader, more worldwide outlook. In this way, “*arts and cultural organizations*” can both reinforce the importance of the arts and the culture and maintain their own significance in society.

One of the remarkable cultural organizations is museum, it plays a vital role in building a knowledge-based society, which in turn contributes to the economy, because museums are institutions that aim to produce knowledge and/or represent people, their cultures and histories. The International Council of Museums (ICOM) has perfectly acknowledged the social role of museums in our society, where on August 24, 2022, the Extraordinary General Assembly of ICOM has approved the proposal for the new museum definition “*A museum is a not-for-profit, permanent institution in the service of society that researches, collects, conserves, interprets and exhibits tangible and intangible heritage. Open to the public, accessible and inclusive, museums foster diversity and sustainability. They operate and communicate ethically, professionally and with the participation of communities, offering varied experiences for education, enjoyment, reflection and knowledge sharing.*” [1]. The ultimate goal is to deliver information to a large audience and to achieve justice for everyone's right to education and culture. Museums represent important civilized and cultural interfaces for their countries that express their potential and creativity, especially after the concept of museums has changed in recent years as a result of the tremendous efforts made by the specialists in museum affairs. Therefore, the association of museums with society is considered an important matter. Museums rolesare no longer limited to the acquisition, preservation and display of objects without the presence of any cultural programs or activities, but rather changed to include organizing the necessary educational and cultural programs, whether inside or outside museums, in order to attract the audience with their different social groups, and communicate with them on a permanent and continuous basis in an effort to convey the museum's civilizational, historical, cultural and heritage message. In 2005, the Department of Culture, Media and Sport (DCMS) in UK published a consultation paper [2] stating that museums and galleries “*play an essential role in helping its citizens to understand their place in the world and its heritage and that they connect our past with our present and our future*".

According to Becker (1966) [3], the theatre can play different roles in our society. First, it offers needed break, a chance of a lifetime and a chance to get away into healthful fantasy. Second, theatre presents a necessary intellectual challenge, or a challenge to audience imagination that they will not find in the rest of their lives. And finally, theatre helps as a guide to living.

The library is a cultural organization that has existed in our society since ancient times. It is a place where the interaction between audience and information takes place and aims to meet informational and social needs. Libraries play a crucial role for a nation in preserving its cultural heritage. In the modern age with abundance of information, libraries help the society by maintaining relevant information and publishing it when it is needed [4].

Throughout its history, opera and orchestra have largely demonstrated their social adaptability; a characteristic that made their world immune regardless of the historical contexts, social conditions, economic conditions, political systems and cultural backgrounds that surrounded them. Moreover, the ability of opera and orchestra to be considered a phenomenon of high social value in all the societies, in which they have been presented, has enabled them to communicate or establish a relationship with a broad range of audience kinds [5].

In general, the audience of “*arts and cultural organizations*” can be categorized into five groups, which are: an audience coming frequently, another rarely comes, an absent audience, tourists and finally young audience. These organizations should diversify, widen and attract new audience; the aim of such an action is on the one hand, to increase revenue by attracting a wider audience, and on the other hand to honour their societal role. This brings us to the term “*audience development*”, which remains a challenge for many arts and cultural organizations in the world, because the artistic and cultural offers continue to grow, people have access to a greater variety of hobbies both inside and outside the home and the income available to them increases little when compared to the rising cost of living. The present work aims to identify and compare the different adopted strategies by some “*arts and cultural organizations*” for their “*audience development*”. The involved organizations in this study will be museums, theatres, libraries and music institutions (operas, music house, orchestra, .etc).

However, before presenting this concept within the context of the arts and cultural organizations, it is important to define its basic concept in general, where a possible definition of the concept of “*audience development*” can be given as follows “It is the process of building and expanding an audience for a particular product, service, or organization. It involves understanding the potential audience, identifying their interests and needs, and developing strategies to reach and engage them”. The continuous change and transformation of the roles that the “*arts and cultural organizations*” play in social, political and economic contexts, have highlighted the need for them to act on their relationship with their audience [6]. Practically, “*audience development*” arose as a feedback to this need [7]. The notion of “*Audience Development*” was originally used in arts field in the 1980s [8]. The Arts Council of England defines it as “*Taking a specific action to meet the needs of current and future audiences, and helping arts organizations develop relationships with the audience. It consists of marketing, programming, education, customer care and distribution considerations*” (p.423) [9]. This term has been defined also as numerously as the organizations that use it. It can be viewed in two ways, the first one is as the objective to increase audience numbers, and the second is developing audience as an individual [10]. Although, when the term “*audience development*” comes to mind, it is usually the first, effective audience development must go beyond attracting audience as consumers, towards creating a cycle of reciprocal exchange between “*arts and cultural organization*” and their audience, allowing theformer to develop theirpresentations according to the requirements and wishes of the latter. While private “*arts and cultural organizations*” primarily use “*audience development*” as a tool to ensure visitor numbers and maintain their position in the cultural market by creating accessible audiences, state-supported arts institutions must adhere to the policy goals of reaching a diverse audience (i.e. represent society as a whole as much as possible) and provide valuable cultural experiences to their audience [11]. In addition to increasing visitor numbers, audience development strategies can create and enhance a positive image of the arts or cultural organization within a community, connecting itself to the cultural vitality of the society [12]. A published study, shared among European cultural organizations, by the European Cultural Commission [13] acknowledges that “*audience development has to do with different knowledge fields, such as democratization, access, participation, co-creation, organizational innovation, leadership and policies*” (p.275). As a brief understanding of the concept of “*audience development*”, one can define it in this present work as " a strategy that is conducted by an arts or cultural organizationin the aim of increasing its audience quantitatively, reinforcing its relationship with the audience and enlarging the audience".

## 2 Methodology

In order to identify the different strategies applied by“*arts and cultural organizations*” for their “*audience development*”, an exploratory literature review was conducted using the following databases: Google Scholar and Semantic Scholar, in addition to the websites of concernedarts and cultural organizations. The search keywords were “strategies”, “audience development”, “arts organizations” and “cultural organizations”. The references approved for the study were limited to those related tothe four selected kinds of institutions among the “*arts and cultural organizations*”, which are museums, theaters, libraries and music (such as the opera and orchestra). It should also be noted that during the search for some “*arts and cultural organizations*” in France and in Canada, such as the Louvre Museum, the French National Library and the National Library and Archives of Quebec, some references written in French were found and approved. Regarding the Arabic language references, only one reference is adopted which concern the museums audience development strategy in the Kingdom of Saudi Arabia (KSA).

## 3 Results

The results of this comparative review study include mainly the strategies adopted and applied by the above mentioned “*arts and cultural organizations*” for their “*audience development*”, where the strategies used by the same type of selected organization will be identified.

### 3.1 “Audience development” strategies applied by museums

The Petrie Museum of Egyptian Archaeology in UK [14] has developed different strategies for its audience development. First, it was one of the first museums in the world to make its whole catalogue available online. Second, spaces in the museum have been reorganized to be more physically and intellectually accessible to the public. The Petrie Museum also hosts children from schools in London and welcomes scholars from around the world, the new space has evolved into an “educational lab” where visitors can test the advanced technological tools such as “*3D imaging technologies*” being developed in the aim of its “*audience development*”. And finally, the regular exhibitions organized by the museum was a key factor in its “*audience development*".

One of the strategies proposed for contemporary art audience development is the involvement of public art museums in the concept of “*constructive museum*” [15], that means “*acknowledging and empowering the learner as an active participant in the construction of knowledge and meaning*” (p.2), thus the role of the public art museum is reoriented from expert speaker to expert listener.

Tsima (2021) presented some strategies applied by Greek museums in the goal of their audience development. The Cycladic Museum regularly holds temporary exhibitions of modern and contemporary art, where a parallel conversation is held about its ancient collections. The Benaki Museum was amongst Greece's first museums to initiate a specialized educational part. Bassilis & Marina Theocharakis Foundation, as a “*common motif in the genealogy of modern Greek museums*”, offers a variety of events including exhibitions, concerts, lectures and educational activities, in which it uses new digital means and social media facilities for its “*audience development*” [16]. [17] Albuquerque (2018) showed that one of the marketing strategies used by Portugal's museums for their “*audience development*” is cultural and entertainment programs, in addition to attracting tourists.

Lee (2005) [18] showed that, in the “*Jordan Schnitzer Museum of Art at the University of Oregon*”, developing art education outreach materials from a multicultural perspective promotes audience development the museum.

The museums of Finnish Lapland adopted the strategy of “*museum pedagogy*” or “*museum education*”as the corner stone of their “*audience development*” [19]. Through this strategy, the museums facilitate, encourage and support the visitor experience and activate interpretation and creativity. Theyinvolve human rights, equality, democracy, sustainable environment and tolerance. In addition, theyoffer methods of practices in artistic education and public works, cooperate with other institutions, and support school programs. The methods used by this strategy are miscellaneous.

Ahn (2004) [20] presented the corporate sponsorship of Universeum Museum “*The Gothenburg based natural science museum in Sweden*” as a strategic choice for its “*audience development*”, where it is demonstrated that the various cultural, educational and recreational activities and programs with corporate sponsorship provide the public with entertainment and valuable cultural acquisition and a deep understanding of the diverse fields of arts and culture. Tulliach (2017) [21] showed also that museum partnerships are one of the applied strategies by some museums which creates networks and attract new audiences. Theresearch gave some case studies of museum cooperation from Bologna (Italy), such as *Istituzione Bologna Musei*, which gathers the municipal museums of the city; the *Polo Musealedell’Emilia-Romagna*, which connects the state museums from the same Italian region; and the ICOM Emilia-Romagna Regional committee, which assembles museum staff members from the entire region, in addition to the cooperation between the Archaeological Museum of Bologna and the Rijksmuseum van Oudheden (Leiden), where the both share the same archaeological project and several mutual loans. Another example of “*museum partnerships*” isSharjah Museums Department (SDM) partnership in the United Arab Emirates (UAE) [22].

Indian museums use five strategies for audience development [23]. First one is audience segmentation approach by dividing it as a function of intention, age, aims, professions and ethnicity. Second one is adopting a visitor-centered approach as the motto of a successful museum. Third strategy is creating communication modes, through making museum signage, captions, wall text, and marketing materials speak the language of the audience. The fourth one is creating simple sensorial experiences. Indeed, stimulating the five human senses through simple experiences can have an impact on the visitor. The last strategy of Indian museums is partner complementary organizations; It is a novel idea in India.

In Egypt, the Alexandria National Museum implements public development programs to raise awareness in the community for the preservation of cultural heritage [24]. The museum offers science courses for school children. There is also a “*Junior Archeologist*” program for children over 5 years old. The museum organizes cultural events by showing films on history and heritage, as well as workshops and training in the preservation of artifacts and other objects and sites to raise awareness of how to treat tourists. The museum also organizes joint workshops or conferences with the Bibliotheca Alexandrina. It also organizes training courses for students of the Faculty of Tourism and Hospitality.

In Italy, MAXXI is an example of improving the social role of museums based on long-term relationships with social actors, as this situation leads to sustainable development of audiences in museums [25], MAXXI has been shown to have important projects “*Il Mio Iran (My Iran), Il Museotra I banchi di scuola and MIXT – Musei per tutti*” which represent different approaches and strategies for “*audience development*”, the strategies are based on two main areas of action to diversify the audience, the first It is active participation, as the museum focuses on the participatory factor, including and engaging new audiences. The second is collaboration, communication and innovative partnerships, where the museum engages in partnerships or collaborations to reach new target audiences.

In Austria [26], ambitious projects for young people have been launched by Buro fur Kulturvermittlung (a non-governmental organization), working with trainees as a new audience for the museums. The Regional Museum of Carinthia has created some innovative programs for athletes who are traditionally non-museum attendees, another activity conducted by the museum targeted the young audience by organizing a science exhibition in the museum in cooperation with a local school, where the students actively participated in this activity and were part of it and contributed to its success.

One of the strategies that contribute to “*audience development*” is audience research [27]. In this study, “*Audience Research*” is presented to enable museums in the United States to expand services, programs, and exhibitions to encourage visitor participation and enjoyment. In this context, the Minnesota Historical Society is focusing on the impact of a new facility by providing better facilities for gallery space and visitor services, and the Art Institute of Chicago has sought a diverse audience, expanding its offerings by offering programs and classes for teens, families, college students, and the elderly. There is also greater communication and engagement among African Americans, while the Montclair Museum of Art conducts its “audience research” by surveying visitors at its many exhibitions it curates each year, organizing free programs and lectures for students, scholars, educators, and families.

In United Arab Emirates (UAE), a recent survey study [28] showed that expatriate groups are an important determinant of audience attendance of museums; therefore, the UAE government has launched the extension of the long-term visa program, introduced in 2019, to include more categories of expatriates, which could contribute to audience development in museums.

In general, digital technology provides a tremendous opportunity for mostmuseums by allowing them to be ubiquitous, to exist in diverse forms that meet the needs of different visitors, to interact with new audiences and, above all, to build relationships that are more important than a traditional museum visits. In addition, during the confinement of Covid-19, digital technology is more important than ever as a tool of communication between arts organizations and their audiences, where a huge boost in digital activities was given to the public at trade fairs [29]. Cerquetti (2016) [30] confirmed the fundamental role of ICTs “*Information and communication Technologies*” for museum modernization in Europe, both for the enhancement of service quality and the attraction of new audiences, thus encouraging edutainment, interaction, interactive experiences and narrative atmosphere. Alfandari (2015) [31] indicated that in the case of Louvre museum's audience, “*with platforms such as Facebook, Twitter, YouTube, Pinterest, or Instagram, for example, the Louvre can reach new, younger, and more remote audiences (Europe, the Americas, etc.) who may never have visited the museum but take an interest in what's happening there*”. Talbot (2019) [32] indicated that Louvre uses digital technology to facilitate the access to all art collections of the museum, to target new audience and to digitalize the art pieces which allow the general public to access the entirety of the museum's collection, because the museum doesn't exhibit all its owned pieces. Digital technology also enables the British Museum to increase its audience [33] (nearly five million followers on social media), it has turned to Hootsuite to help implement its multi-channel publishing strategy and enable its social team to interact frequently and effectively with its audience. Research activities carried out in Greece, Portugal and Italy within the Mu. SA “Museum Sector Alliance” project [34] concluded that “*Today staff highly skilled in digital skills are crucial in order to help museums use new technologies to multiply opportunities for exchange, accessibility and participation for audiences*” (p.57). The Louvre Abu Dhabi applied commercial insight marketing technique in selecting media, strategic messages through images, questions and creating anticipation for the audience. This study indicates that through social media, Louvre Abu Dhabi is able to market its activities and events and promote the UAE to a global audience online. By providing digital platforms to engage audiences and gain feedback, social media helps the Louvre Abu Dhabi fulfill its cultural function of spreading intercultural understanding and tolerance in the UAE [35].

In Canada, the Montreal Museum of Fine Arts enlarged its audience through the exhibitions that have captured the public, unifying projects on the occasion of the 375th anniversary of Montreal, a growing range of cultural and educational activities, the development of innovative initiatives in the field of well-being and the newly expanded spaces of “*Michel de la Chenelière*" international workshop for education and art therapy and participation in a wide range of educational, cultural and health activities [36].

In Japan, the Tokyo National Museum adopted three tips [37], the first is to change events from educational to entertaining (like films), the second is to change marketing tools from paper based to digital based, and finally, the third is holding events regularly to retain their interest.

In summer 2019, the British Museum held the largest exhibition of Manga [38] ever to take place outside of Japan; this initiative was a significant opportunity for the museum to attract new audience to its historical exhibitions. There was a lot of modern technical equipment as the curators try to appeal to the younger audience. For example, there was a digital display of a bookstore, a digital experience based on the adventure of Professor Munakata's Hoshino Yukinobu Museum in the UK, and a humorous show Takaoka, the oldest Manga library in Tokyo. For a themed outing, visitors can “enjoy the Manga” at the special photo booth.

Gompertz (2004) [39] noted that developing new audiences is central to four of Tate's strategic goals, namely: developing new audiences both inside and outside of showrooms. Finding new ways to reach new audiences; Visitor audits to inform Tate's approach to improving future visitor care and creating new paths for Tate through their programs - large and small, some of these projects are: ‘Late at Tate Britain’, ‘Raw Canvas' and ‘Turner and Venice’, which have developed and reached new audiences for the Tate Museum in the UK.

The Metropolitan Museum of Art in New York adopted the multicultural audience development Initiative which reflects the museum's founding mission of educating and inspiring by reaching all of its audiences [40]. For example, it celebrates Hispanic and Latin American culture through social events, however its audience development is geared towards other cultures though events such as Chinese New Year, LGPT party and women's rights events, etc.

The museums authority in the Kingdom of Saudi Arabia launched, in September 2021, its strategy, in light of which it will work to develop the museum sector in the Kingdom with all its components and organizational and operational tracks, and to support and empower practitioners and investors in it, in order to achieve the goals of the Ministry of Culture and the goals of Saudi Vision 2030, in its cultural aspects. The strategy stipulated the establishment of national museums as inspiring destinations and catalysts for the cultural and societal participation of the public, including citizens, residents and tourists. The strategy will contribute to the development of existing museums such as the National Museum of Saudi Arabia and Al Masmak Palace Museum, and will also create a large number of museums around the Kingdom's regions [41].

### 3.2 “Audience development” strategies applied by theatres

The Royal Shakespeare Company (RSC) is a major British theatre company in UK. The RSC plays regularly in London, Stratford-upon-Avon, and on tour across the UK and internationally. Its purpose is to Promote Shakespeare's plays to a truly diverse audience and their relevance to today's society [42]. Therefore, RSC adopted audience segmentation for their audience development, where it developed a short and medium-term plan to develop new relationships and understanding of new audiences, using values Based Networking (VBN) that creates a suitable environment to gain real insight into the changes RSC needed to make as a company [43].

The “*Comédie-Française*" or “*Théâtre-Français*" targets different audiences in its audience development strategies. For young people, the Office of Young Readers and Authors was founded on the principle of discovering classical and contemporary texts. A citizen project “*Allonsenfants de la culture!*” created to allow students of “*GrandesEcoles*” discovering theater and opera during a public experience that combines performances, visits to institutions and meetings with artists. Moreover, “*Comédie-Française*" offers several ticket formats for young people to facilitate their access to the theater, both individually and as a family or group. For school children, “*Comédie-Française*” has expanded its social and geographical scope by developing partnerships with schools in priority areas of education, technological and vocational schools and schools located in rural areas. “*Comédie-Française*" also does specific reception work with young refugees educated in French secondary schools or pursues the “*AccueilRéfugiés*" program to help them immerse themselves in French theater culture. For the university audience, “*Comédie-Française*" partners with about forty institutions of higher education (universities, major schools and preparatory classes). It also pays special attention to teachers, recognizing the key role they play in taking the stage to young people, and finally providing free digital resources to all audiences on its website [44].

[45] Hansen (2015) presented and analysed the results of an “audience development” project that the author carried out in Central Denmark Region in the period 2010–2012 using the “Theatre Talks” method [46,47] involving 18 theatres. Various audiences were invited into the theatre to participate in a “Theatre Talk”, thus allowing them to share the experience of going to the theatre with each other after the performance. The project aimed to build relationships with new audiences and to gain a greater insight into their experience of theatrical events. Lindelof and Hansem (2015) [48] also recommended this project to two Swedish-Danish performances: “*A Thousand Rooms of Dream and Fear*” and “*Bastard*” for their audience development strategy.

Lithuanian theatres, such as“ Kaunas National Drama Theatre” and “Kaunas National Drama Theatre”, in their audience development strategies, targets their loyal audiences, who usually are invited to join theatre clubs, offered various membership programs, long term subscriptions for theatre tickets, and newsletters [49].

IN UAE, besides facilitating residency ofexpatriate groups as a theatre audience development strategy, there is alsoanother strategy, which is engaging youth in cultural participation, e.g. lessons in the arts theatre, where it was found that arts theatre lessons increase the chances and frequency of attending the theatre, such as “Dubai Community Theatre” [28].

### 3.3 “Audience development” strategies applied by libraries

The world of libraries is currently experiencing a series of revolutions: the digital revolution, revolution in principle and a revolution in practices. Crossed by these upheavals, libraries are committed to redefining their missions in order to adapt their service offer to the needs of these “new users". In the face of declining attendance, major great libraries in the world are now striving to reinvent itself. In the face of evolving practices and uses, it is developing its service offering to the public, with an active policy of restructuring the library's display and spaces, ant it will attempt to meet the challenge of increasing and diversifying its audience.

The French National Library (FNL) has adopted different strategies for its audience development [50]. First, it has chosen to specialize its resources according to the typology of audiences. This segmentation makes it possible to personalize action plans according to the specific needs of each and to respond to them with composite offers of different services and activities of the FNL by the implementation of partnership and promotion operations. Second, the FNL promotes its offers at targeted trade fairs and forums such as Paris Book, Mans’Art, Musicora, Paris Games Week, Art History Festival, Sorbonne University Back-to-School Forum, Montreuil Book and Youth Press Fair. Third, it personalizes relations with the public through the operational deployment of the CRM “*Customer Relationship Management*” tool which the FNL acquired in 2016 to centralize its contacts and organize targeted communication with its public. Fourth, the FNL offers an interface for its website in French, English and Spanish. Finally, the FNL builds close and lasting links with the public through academic partnerships.

The Library of Congress (LOC), the national library of USA, and the largest library in the world, offers different programs as strategic actions for its audience development [51]. For example, “*World Digital Library*” program, which provides online free access to manuscripts, rare books, maps, photographs, and other important cultural documents from all countries and cultures, in Arabic, Chinese, English, French, Portuguese, Russian and Spanish, “*John W. Kluge Center*” program which gives scholars access to the Library's collections & engaging them in conversation with public policy leaders, free concerts' performances by extraordinary artists in classical, jazz, folk & other genres and “*Center for the Book*” program which promotes books, reading, libraries & literacy through its national center in Washington & its affiliated centers across the country.

The British Library (BL) [52], national library of the United Kingdom, offers practical information online about the library in PDF format in 18 different languages. It attempts to engage wider audience with its memorable cultural experiences, where its challenge over the coming years is to help more people discover and enjoy its exhibition and events program, in addition it plans to expand and improve its galleries and share its collections with wider audiences looking beyond the UK to ensure audiences overseas have more opportunities to appreciate its collections, the British Library plans also to develop an increases program of loans, touring exhibitions and digital collaborations with public that open its collections to new audiences, both across the UK and internationally. Targeting new audiences, the British Library Labs project gives the opportunity to everyone to experiment with the library's digital collections, including deploying computational research methods and use its collections as data.

The National Library and Archives of Quebec” BAnQ” offers an interface for its website in French and English [53]. Its desire to open up to audiences not accustomed to libraries tends to make communication more difficult. One of the solutions could be to move away from its registers of language, in order to more easily establish contact with the target audiences. The BAnQ's “*youth space*” or “*espacejeune*”, designed in the form of a treasure map, illustrates here a real communication strategy targeted towards a specific type of public. The trend is therefore towards the diversification of communication registers, towards an image more dynamic, more modern and less austere institutions. Another strategy of audience development that is adopted by BAnQ is academic partnerships in the frame of the project of “Service québécois du livre adapté” which provides perceptually impaired users with packages and services adapted to their needs in terms of information, education, culture, research and entertainment.

The Royal Library of the Netherlands “*Koninklijke Bibliotheek (KB)*” tries in its strategic plan (2019–2022) [54] to attract all types of readers, it is therefore aware that people need different media at different times; For print or digital readings, audiobooks or plain texts, it is aimed at avid readers and people who consider listening as the new form of reading and strive to make its services user-friendly and engaging. The Koninklijke Bibliotheek, together with the public libraries, provides a continuous reading line that corresponds with the reading education and also forms a challenge for advanced readers. In addition, it invests in its development and in working in national and international networks, including the network of public libraries.

### 3.4 “Audience development” strategies applied by music institutions

Sigurjonsson (2010) [55] noted that with respect to musical institutions, ideas of the listener as a consumer object demanding greater access and more comfortable musical participation seem to be the main tenets of mainstream audience development theory.

Tajtáková and Arias-Aranda (2008) [56] conducted a survey in Bratislava, the capital of the Slovak Republic, among university students, mostly between the ages of 18 and 26, about their attendance and intention to attend opera and ballet performances offered by the Slovak National Theatre, and concluded that university students in general could be considered an “easy target” in audience development strategies for opera and ballet.

From a case study regarding the audience development of chamber music attenders at Music in the Round's inaugural and second year national tours in UK [57], it is found that developing a successful audience requires a strong commitment to working outside the usual channels of marketing traditional arts. The willingness to try new approaches and make innovative ads are also key factors. Actually, the “Music in the Rounds” regularly hosts concerts by partners including Sheffield Music Academy, Sheffield Music School, the University of Sheffield, the Abbeydale Singers, and Sheffield Young Singers, a strategy that provides audience development opportunities and continues to build positive relationships [58].

Swanevelder (2018) [59] explored audience development strategies for South African University Symphony Orchestras, which aim to ensure stability or growth in attendance, and concluded that there are different audience development strategies for symphony orchestras, such as audience engagement through programming, music education within the school curriculum, audience segmentation, digital technology for communication, branding and customer subscription instead of single ticket.

Savage (2015) showed how the Singapore Symphony Orchestra has combined audience development strategies into a cohesive branding and marketing strategy to harvest its profits.

Jiang (2019) presented the strategies adopted by IOrchestra “*a big touring project from the Philharmonia Orchestra (London, UK)*” for audience development, where it has been shown that, besides using technology to outreach and engage, IOrchestra demonstrates the value of a “whole family” or “whole community” approach rather than limiting it to school based projects.

The Royal Opera House, located in Covent Garden Center, is the UK's leading provider of opera and ballet performance. Its facade has been extensively restored, the facade invisible from the district's central market square, and the perceived exclusivity and elitism generally associated with its art forms also pose a challenge. However, social inclusion and audience development initiatives nurturing a new generation of opera and ballet enthusiasts have emerged as important results, as the house's open door policy to daily visitors as well as live broadcasts of ongoing opera and ballet productions elsewhere generate interest in seeing the building from the inside [60].

A new brand management paradigm, named “community paradigm”, has been discussed [61] giving an example of the “3e Scène”, the Paris Opera 3rd stage, which is a fully digital creative platform, and appears as a good illustration of the new community branding paradigm. The name of the project refers to the fact that the opera company was already performing on two actual stages in the heart of Paris: The Palais Garnier and the Opéra Bastille. The third scene “3e Scène” takes place online, in the aim of reaching an audience beyond the ordinary opera audience, where the contemporary language of “3e Scène" reduces distances between audience and opera.

## 4 Discussion

In Table 1, we give the number of references used to analyse audience development in the studied arts and cultural institutions, as we note that most of the references are concentrated within the framework of the study of museums (27 references), while the number of references for other organizations (theatre, library and music institutions) are less than 10 references for each of them.

**Table 1:** Number of used references for each studied arts and cultural organization.

Organization	Museum	Theatre	Library	Music
Number of references	27	8	6	9

We also give in Table 2 the names and locations of the arts and cultural organizations that were targeted in the study.

**Table 2:** Name of the arts and cultural organizations that are cited in this paper.

Type of organization	Name	Location
Museum	The Petrie Museum of Egyptian Archaeology	London-UK
	The Cycladic Museum	Athens-Greece
	The Benaki Museum	Athens-Greece
	Bassilis& Marina Theocharakis Foundation	Athens-Greece
	Jordan Schnitzer Museum of Art at the University of Oregon	Oregon-USA
	Universeum Museum	Gothenburg- Sweden
	Istituzione Bologna Musei	Bologna-Italy
	Polo Musealedell’Emilia-Romagna	Bologna-Italy
	Archaeological Museum of Bologna	Bologna-Italy
	Rijksmuseum van Oudheden	Leiden-Netherlands
	Alexandria National Museum	Alexandria-Egypt
	MAXXI	Rome-Italy
	The Regional Museum of Carinthia	Carinthia-Austria
	Montclair Museum of Art	New Jersey-USA
	The British Museum	London-UK
	The Louvre	Paris-France
	Louvre Abu Dhabi	Abu Dhabi-UAE
	The Sharjah Art Museum	Sharjah-UAE
	The Montreal Museum of Fine Arts	Quebec-Canada
	The Tokyo National Museum	Tokyo-Japan
	Tate	London, Cornwall, Liverpol-UK
	The Metropolitan Museum of Art	New York-USA
	The national museum of Saudi Arabia	Riyadh-KSA
	Al Masmak Palace Museum	Riyadh-KSA
Theatre	The Royal Shakespeare Company	Stratford -UK
	Comédie-Française	Paris-France
	Theatre network	Midtjylland-Denmark
	Kaunas National Drama Theatre	Kaunas-Lithuania
	Kaunas State Puppet Theatre	Kaunas-Lithuania
	Dubai Community Theatre	Dubai-UAE
Library	The French National Library	Paris-France
	The Library of Congress	Washington-USA
	The British Library	London-UK
	The National Library and Archives of Quebec	Montréal-Canada
	The Royal Library of the Netherlands	The Hague- Netherlands
Music	Slovak National Theatre	Bratislava- Slovak republic
	Music in the Round	Sheffield, UK
	University symphony orchestra	Potchefstroom-South Africa
	The Singapore Symphony Orchestra	Singapore
	IOrchestra	London-UK
	The Royal Opera House	London-UK
	Opéra de Paris	Paris-France

**Fig. 1:**
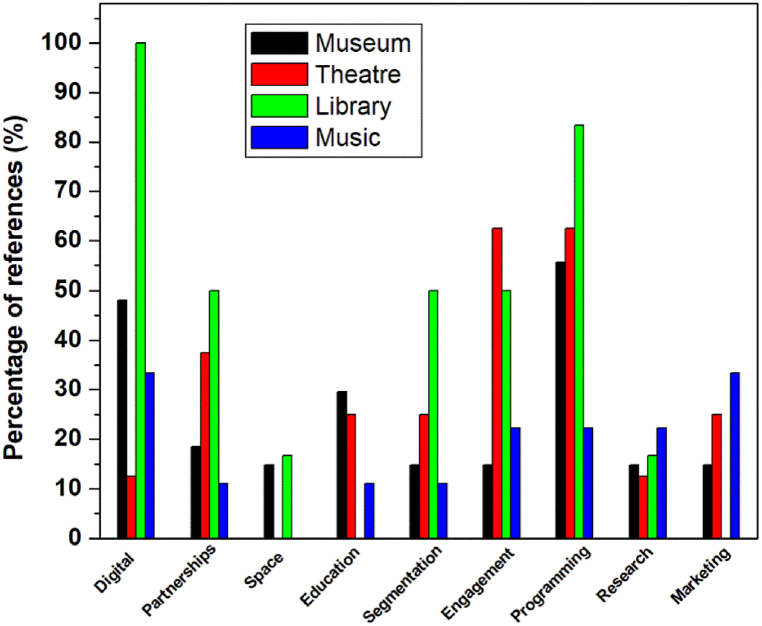
Diagram of percentages of references related to the nine strategies of the four studied types of arts and cultural organizations.

To summarize all the identified strategies that are applied by the four types of the arts and cultural organizations for their audience development, Table 3 shows the list of the nine found strategies in the literature attributing them to the type of the organization, where the number of cited articles (N) are provided with its percentage (%: calculated by taking into account the total number of references for each type of organization given in Table 1), these data are illustrated also in Fig. 1. Adding to these nine strategies another approach, not listed in the table, and which is adopted by UAE government to increase audience attendance to its arts and cultural organizations, it consists of changing residency rules for the expatriates in UAE to extend their stay in the country, because they are potential audiences for these organizations.

**Table 3:** Identified strategies applied by arts and cultural organizations and the corresponding number of cited articles in this study.

Strategy		Organization							
		Museum		Theatre		Library		Music	
		N	%	N	%	N	%	N	%
1	Digital Technology	13	48.1	1	12.5	6	100	3	33.3
2	Partnerships	5	18.5	3	37.5	3	50	1	11.1
3	Physical space development	4	14.8			1	16.7		
4	Education	8	29.6	2	25			1	11.1
5	Audience segmentation	4	14.8	2	25	3	50	1	11.1
6	Public engagement	4	14.8	5	62.5	3	50	2	22.2
7	Programming	15	55.6	5	62.5	5	83.3	2	22.2
8	Audience research	4	14.8	1	12.5	1	16.7	2	22.2
9	Marketing	4	14.8	2	25			3	33.3

For the Digital Technology strategy, social media platforms like Facebook, Twitter, and Instagram have become powerful tools for connecting arts and cultural organizations with their audiences. For example, the Metropolitan Museum of Art in New York City uses social media to promote its exhibitions and events, showcase its collections, and engage with its followers. Through social media, the museum has reached a younger audience and built a community of art enthusiasts.

In the Audience segmentation strategy, this can be driven using the Personalization approach, which involves tailoring programs and experiences to individual audiences' specific interests and needs. The Royal Shakespeare Company in the UK uses a data-driven approach to personalize its marketing and outreach efforts. The company can create targeted campaigns that resonate with different audience segments and increase engagement by analysing audience data.

Within the Public engagement strategy, diversity and inclusion are pertinent for the arts and cultural organizations to reach out to diverse audiences by programming events that reflect different communities' interests and cultural backgrounds. They may also provide accessible performances and events for people with disabilities. For example, the Tate galleries in the UK have a strong focus on technology, with a range of online content that engages audiences in new ways. They also have a partnership program that includes collaborations with other cultural organizations and businesses, and they offer accessible performances and events for people with disabilities.

As one can notice form the figure, the museums apply all the nine strategies, but they depend mainly on two strategies: Digital Technology (48.1%)and programming (55.6%). Theaters don't care about increasing space, and they rely mainly on three strategies: Partnerships (37.5%) public engagement (62.5%) and programming (62.5%). The Libraries do not approach in their strategies towards developing audiences by increasing space, audience research studies and marketing, while they fully use digital technology (100%), they are highly interested in programming (83.3%) and they equally (50%) use partnerships, audience segmentation and public engagement. The music institutions apply all strategies except of increasing their physical spaces, with a nearly equal interest.

## 5 Conclusion

Nine strategies of audience development, that concern four types of arts and cultural organizations (Museums, Theatres, Libraries and Music institutions), emerged from the analysis of the articles obtained from the literature and the websites of the organizations. Theses found strategies are: Digital Technology, Partnerships, Physical space development, Education, audience segmentation, public engagement, audience research and marketing. The results showed that digital technology is largely used by museums, libraries and music institutions, while the theatres essentially take care of public engagement and programming. The strategies of all the four types of the organizations rely on partnerships, audience segmentation, public engagement, programming and audience research studies. The extension of the physical space concerned only museums and libraries.

The results show the many efforts that arts and culture organizations are making to adapt to current technological, social, and economic challenges, and how these efforts are being of interest to the academic community. Arts and cultural organizations implement audience development strategies to enhance public participation and provide access to culture, as well as create new management processes to support these organizations in the cultural and entertaining market. This has the potential to enhance the reach of audience development strategies within society. In this way, they can improve the relationship between organizations and certain community groups, while raising awareness among their audience.

As a result of the conducted literature review and the identification and analysis of the nine different strategies that have emerged, a comprehensive understanding of the scholarly debate on audience development within arts and cultural organizations (in English, French and Arabic) has been gained.

## 6 Implications for management

This article provided an overview of the academic debate on audience development within four types of arts and cultural organizations (museums, theaters, libraries and music institutions). It indicates that academic interest has arisen through the efforts of these organizations to get closer to their community and audiences through better knowledge of different audience groups and new management strategies. This article tried also to cover questions about how arts and cultural organization can better be managed in a fast-changing world at technological, social end economical levels.

Our Findings showed the existence of different nine categories (Digital Technology, Partnerships, Physical space development, Education, audience segmentation, public engagement, audience research and marketing.) of strategies used by arts and cultural organizations in their management of audience development. The strategies were found to be used relatively by the four studied organizations, for example, when the most used strategy by libraries is digital technology, the museums use mostly programming strategy, in addition some categories aren't applied by all the studied organizations, for example space development strategy is not applied by both theatres and music institutions.

Therefore, the given results in this article can inspire different arts and cultural organizations especially that are not cited in this work, to adapt their management strategy regarding audience development, depending on the type of organization. Our results also can enlighten the arts and cultural organization in the developing countries through the inspirational management strategies applied by the cited organizations around the world.

Through this work, we suggest that arts and cultural organizations, through their management of strategies related to the audience development, adopt in-depth audience research studies, because there is a lack of such studies from one hand, and the audience research coincides for the benefit of both arts-cultural organizations and the public. By recognizing the interests and dedication of visitors, arts and cultural institutions can change or modify various aspects of their operations to encourage visitor participation and increase the visibility and educational benefits of the targeted organization and the various opportunities that are offered, this type of research studies is a key factor in the organization's management strategy.

## Author contribution statement

All authors listed have significantly contributed to the investigation, development and writing of this article.

## Data availability statement

Data will be made available on request.

## Declaration of competing interest

The authors declare that they have no known competing financial interests or personal relationships that could have appeared to influence the work reported in this paper.
